# Retrograde type A aortic dissection during or after thoracic endovascular aortic repair: a single center 16-year experience

**DOI:** 10.3389/fcvm.2023.1160142

**Published:** 2023-07-21

**Authors:** Guo-quan Wang, Ya-fei Qin, Shuai-tao Shi, Ke-wei Zhang, Shui-ting Zhai, Tian-xiao Li

**Affiliations:** ^1^ Department of Vascular Surgery, Zhengzhou University People’s Hospital, Henan Provincial People’s Hospital, Zhengzhou, China; ^2^ Henan Provincial Neurointerventional Engineering Research Center, Henan International Joint Laboratory of Cerebrovascular Disease, and Henan Engineering Research Center of Cerebrovascular Intervention Innovation, Zhengzhou, China; ^3^ Department of Cerebrovascular Disease and Neurosurgery, Zhengzhou University People's Hospital, Henan Provincial People’s Hospital, Zhengzhou, China

**Keywords:** aortic dissection, endovascular repair with stent graft, retrograde type A dissection, oversizing ratio, risk factors

## Abstract

**Objective:**

This article aims to investigate the incidence rate of retrograde type A aortic dissection (RTAD) and the risk factors of RTAD in relation to thoracic endovascular aortic repair (TEVAR).

**Methods:**

Patients with thoracic aortic disease who underwent TEVAR at Henan Provincial People's Hospital from January 2004 to December 2019 were enrolled in the present research. The risk factors associated with RTAD following TEVAR using univariate and multiple logistic regression analyses.

**Results:**

During the study period, A total of 1,688 TEVAR patients were included in this study, and of these, 1,592 cases were included in the type B aortic dissection (TBAD) group, and 96 cases were included in the non-TBAD group. There were 1,230 cases of aortic dissection and 362 cases of aortic intramural hematoma and/or penetrating ulcer in the TBAD group. The non-TBAD group included 68 cases of thoracic aortic aneurysm, 21 cases of thoracic aortic pseudoaneurysm, and seven cases of congenital aortic coarctation. The overall incidence rate of RTAD was 1.1% (18/1,688) in patients, all of which occurred in the TBAD group. The cohort comprised 18 RTAD patients with an average age of 56.78, consisting of 13 males and 5 females. Among them, 13 individuals exhibited hypertension. Ten instances happened within the TEVAR perioperative period, including two cases during the surgery, six cases occurred within three months, two cases occurred after one year, and the longest interval was 72 months following TEVAR. TEVAR was successfully implemented in 17 patients, while the operation technique was temporarily altered in one case. The new entry position for RTAD was identified as the proximal region of the stent graft (SG) in 13 patients, while in five cases, the entry site was more than 2 cm away from the proximal region of the SG. 17 cases were at the greater curvature of the aorta, and one case was at the lesser curvature. Multivariate logistic regression analysis revealed that the SG oversizing ratio is a relevant risk factor for RTAD. However, ascending aortic diameter, aortic arch type, SG type, and anchored region were not directly related to the occurrence of RTAD.

**Conclusion:**

RTAD is a rare yet catastrophic complication. It could occur both during the procedure, early and late postoperative periods. Maintaining an appropriate SG oversizing ratio is crucial to minimize the risk of RTAD.

## Introduction

The development of thoracic endovascular aortic repair (TEVAR) represents a cornerstone in the current treatment of thoracic aortic diseases due to the established features of minimal invasiveness and promising therapeutic effects (1). Continual advancements in surgical techniques, coupled with the evolution of stent graft devices, have significantly contributed to improved clinical outcomes and expanded the range of clinical indications (2). In particular, the TEVAR has demonstrated favorable medium and long-term results and was reported as a class I recommendation for complicated type B aortic dissection (TBAD) in the European Society of Cardiology guidelines and Vascular Societies guidelines (3). Remarkably, although TEVAR boasts high success rates, retrograde type A aortic dissection (RTAD) remains a critical vulnerability and a significant challenge in the field (4, 5). As reported in previous literature, the prevalence of RTAD varies from 2% to 12%, with mortality rates exceeding 40% (2, 6–8). RTAD could occur immediately, intraoperatively, perioperatively, or during follow-up. Given its catastrophic consequence, early detection and prevention of risk factors of RTAD are of paramount importance. RTAD may be linked to the lesions of the aortic wall, such as heritable connective tissue disorders, wall edema in the acute stage, radial force, and device oversizing (9). Moreover, both the natural progression of the disease and potential iatrogenic injuries resulting from endovascular manipulation of the arch could contribute equally to the occurrence of RTAD (7). The specific risk factors associated with RTAD continue to be a subject of debate, as previous studies have yielded conflicting findings. Some researchers have hypothesized that the use of a proximal bare stent, aimed at enhancing stent graft fixation within the aortic arch, could potentially elevate the risk of RTAD (10–12). Nevertheless, recent studies have concluded the conflicting findings (9, 13). In the present study, we present our experience with RTAD following TEVAR in patients with TBAD and other thoracic aortic disorders, intending to identify the risk variables for RTAD that will allow the clinician to reduce this fatal complication. Furthermore, these findings will improve our capacity to counsel patients undergoing TEVAR for thoracic aortic disorders about surgical risk and long-term outcomes.

## Methods

### Cohort

In the present retrospective study, patients with thoracic aortic disease including dissections, intramural hematomas, penetrating ulcers, aneurysms and coarctations were enrolled at the Department of Vascular Surgery, Zhengzhou University People's Hospital, Henan Provincial People's Hospital from January 1, 2004 to December 31, 2019. The study complied with the Declaration of Helsinki and was approved by the Ethics Committee of Henan Provincial People's Hospital, People's Hospital of Zhengzhou University. Inclusion criteria: (I) All patients who underwent TEVAR for any indication; (II) The participants with complete clinical data. Exclusion criteria: (I) Patients with incomplete imaging data and definitive diagnosis; (II) Patients accepted conservative treatment without TEVAR.

### Surgical techniques

The surgical techniques were established based on preoperative computed tomography angiography (CTA). All TEVAR procedures adhered to standardized protocols for TEVAR (14, 15). The stage of TBAD and timing of surgery was defined as an acute stage if it was detected within 14 days of symptom onset, subacute stage 14–90 days, and chronic stage after 90 days. If the proximal landing zone measured less than 15 mm from the origin of the left subclavian artery, one of the following procedures was employed to construct an additional proximal landing zone: (I) chimney technique; (II) fenestration techniques; (III) branch stent repair techniques; (IV) coverage of the left subclavian artery (LSA) on purpose, if the right vertebral artery was patent and the left one was not dominant; (V) the left common carotid artery (LCCA) and LSA bypass; (VI) right common carotid artery, LCCA and LSA bypass; (VII) ascending aorta, iliac artery/LCCA bypass. The stent graft is anchored to the healthy vessel wall using the procedures described above. Four models of stent graft device were used: (I) proximal bare stent: Talent and Valiant (Medtronic Vascular, Santa Rosa, Calif), Hercules (Microport, Shanghai, China), Ankura (Lifetech, Shenzhen, China); (II) proximal barbs: Zenith TX2 (Cook Medical, Bloomington, Ind); (III) proximal flared scallops or partially uncovered stents: Gore TAG/C-TAG (W. L. Gore & Associates, Flagstaff, Ariz); (IV) fully covered stent grafts: Castor (Microport, Shanghai, China).

### Follow up

In this study, patients were followed up in the form of telephone interviews, outpatient CTA re-examination, and medical record inquiries until the patient's death or the end of this study. Patients will be followed up at 1, 3, 6, and 12 months after the surgery, with subsequent annual follow-ups until loss to follow-up or mortality occurs.

### Statistical analysis

Statistical analyses were performed with EmpowerStats based on R software (R version 4.2.0). Measurement data were expressed as mean ± standard deviation (SD), and comparison between groups was performed by Student's t-test or one-way analysis of variance (ANOVA). Qualitative data were presented by rate (%), and the intergroup comparison was performed by the Chi-square test. Univariate logistic analysis was used to identify the risk factors associated with RTAD. Logistic multivariate regression analysis to adjust the different potential confounders was performed to determine the effects of oversize ratio and aortic diameter on RTAD.

## Results

### Baseline characteristics

**Figure 1:**
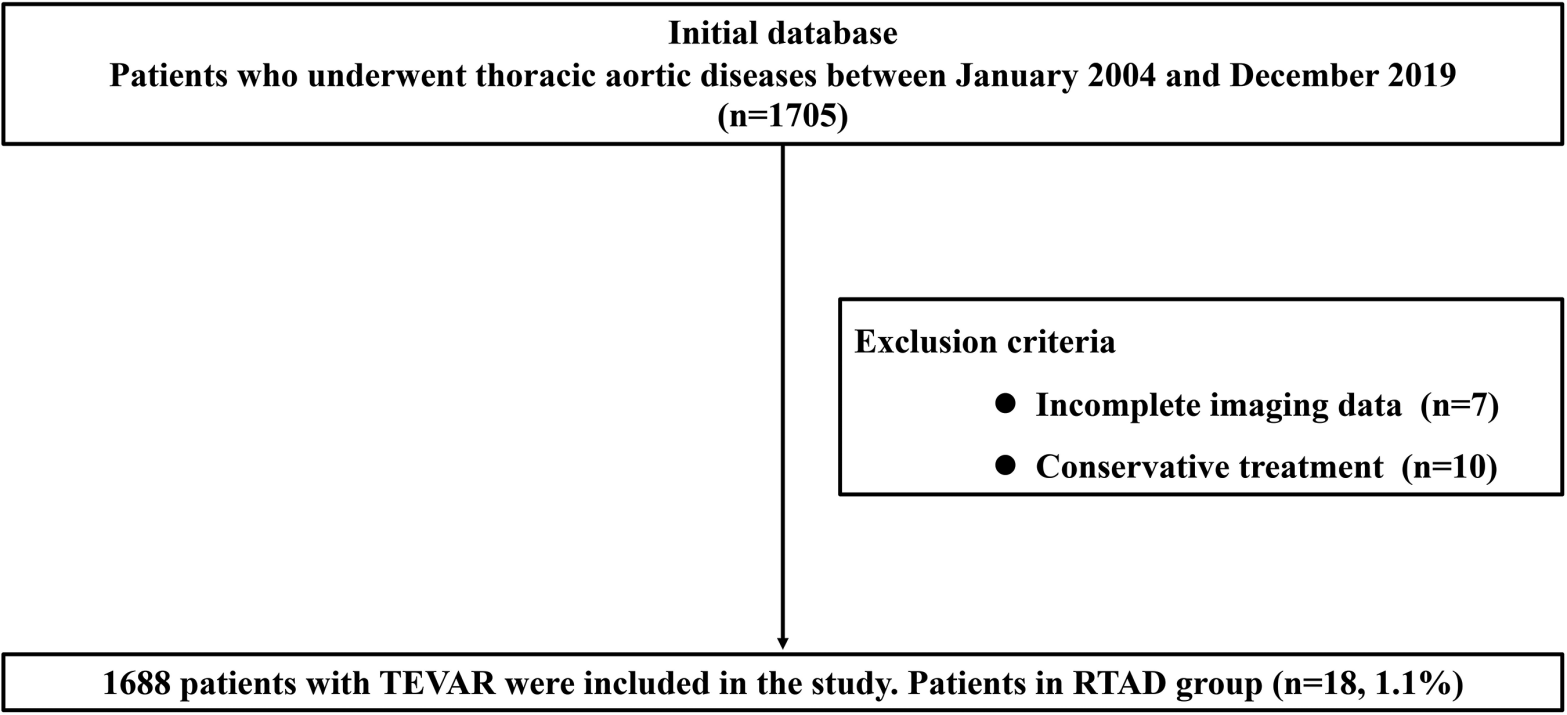
Flow chart of the specific content.

A total of 1,688 TEVAR patients were included in this study, and of these, 1,592 cases were included in the TBAD group, and 96 cases were included in the non-TBAD group. The specific flow chart is shown in Figure 1. There were 1,230 cases of aortic dissection and 362 cases of aortic intramural hematoma and/or penetrating ulcer in the TBAD group. The non-TBAD group included 68 cases of thoracic aortic aneurysm, 21 cases of thoracic aortic pseudoaneurysm, and seven cases of congenital aortic coarctation. A total of 37 patients diagnosed with Marfan syndrome were included in the study, with one case in the RTAD group and 36 cases in the non-RTAD group. The overall incidence rate of RTAD was 1.1% (18/1,688) in patients, all of which occurred in the TBAD group. TEVAR-related complications such as endoleak (8.1%), paraplegia (1.2%), stent graft infection (1.1%) and access injuries (1.0%) were also recorded in the presented research. The basic details of the patients in the RTAD and non-RTAD groups are presented in Table 1.

**Table 1:** Baseline characteristics of TBAD patients who underwent TEVAR.

Variables	Non-RTAD (*n* = 1,670)	RTAD (*n* = 18)
Age	52.48 ± 12.70	56.78 ± 13.36
Gender		
Female	255 (15.27%)	5 (27.78%)
Male	1,415 (84.73%)	13 (72.22%)
Trauma		
No	1,613 (96.59%)	18 (100.00%)
Yes	57 (3.41%)	0 (0.00%)
Connective tissue disease		
No	1,632 (97.72%)	17 (94.44%)
Yes	38 (2.28%)	1 (5.56%)
Hypertension		
No	417 (24.97%)	5 (27.78%)
Yes	1,253 (75.03%)	13 (72.22%)
Diabetes		
No	1,652 (98.92%)	18 (100.00%)
Yes	18 (1.08%)	0 (0.00%)
Cardiovascular and cerebrovascular diseases		
No	1,456 (87.19%)	13 (72.22%)
Yes	214 (12.81%)	5 (27.78%)
Renal insufficiency		
No	1,620 (97.01%)	18 (100.00%)
Yes	50 (2.99%)	0 (0.00%)
Smoking		
No	1,084 (64.91%)	9 (50.00%)
Yes	586 (35.09%)	9 (50.00%)
Pathological type		
TBAD	1,574 (94.25%)	18 (100.00%)
Non-TBAD	96 (5.75%)	0 (0.00%)
Pathological stage		
Acute	1,417 (84.85%)	16 (88.89%)
Chronic	253 (15.15%)	2 (11.11%)
Onset dime (day)	5.00 (3.00–10.00)	3.00 (2.00–6.75)
Surgical producers		
TEVAR	1,301 (77.90%)	11 (61.11%)
(Non-thoracotomy) Hybrid	147 (8.80%)	2 (11.11%)
(Thoracotomy) Hybrid	71 (4.25%)	2 (11.11%)
TEVAR (Fenestration Technique)	53 (3.17%)	1 (5.56%)
TEVAR (Branch Stent Repair Techniques)	47 (2.81%)	1 (5.56%)
TEVAR (Chimney Technique)	51 (3.05%)	1 (5.56%)
Timing of surgical intervention		
Chronic phase	135 (8.08%)	2 (11.11%)
Subacute phase	156 (9.34%)	0 (0.00%)
Acute phase	1,379 (82.57%)	16 (88.89%)
Different types of stents		
Poximal barbs	164 (9.82%)	3 (17.65%)
Fully covered SG	47 (2.81%)	1 (5.88%)
Proximal flared scallops or partially uncovered sStents	465 (27.84%)	3 (17.65%)
Proximal bare stent	994 (59.52%)	10 (58.82%)
Oversizing ratio	11.18 ± 4.77	7.53 ± 3.54
≤10%	860 (51.50%)	14 (82.35%)
11%–20%	750 (44.91%)	3 (17.65%)
>20%	60 (3.59%)	0 (0.00%)
Proximal landing zone^*^		
Zone 0	199 (11.92%)	4 (23.53%)
Zone 1	431 (25.81%)	5 (29.41%)
Zone 2	944 (56.53%)	8 (47.06%)
Zone 3	96 (5.75%)	0 (0.00%)
Arch type^**^		
Type Ⅰ	632 (37.84%)	7 (38.89%)
Type Ⅱ	844 (50.54%)	8 (44.44%)
Type Ⅲ	194 (11.62%)	3 (16.67%)
Diameter of ascending aorta	38.06 ± 4.93	40.56 ± 6.78
<40 mm	1,054 (63.11%)	6 (33.33%)
≥40 mm	616 (36.89%)	12 (66.67%)

TEVAR, Thoracic Endovascular Aortic Repair; SG, Stent Graft; TBAD, Stanford type B aortic dissection.

^*^ Refer to the Ishimaru aortic arch type.

^**^ Refer to the Myla aortic arch type.

### The basic information for RTAD patients

**Figure 2:**
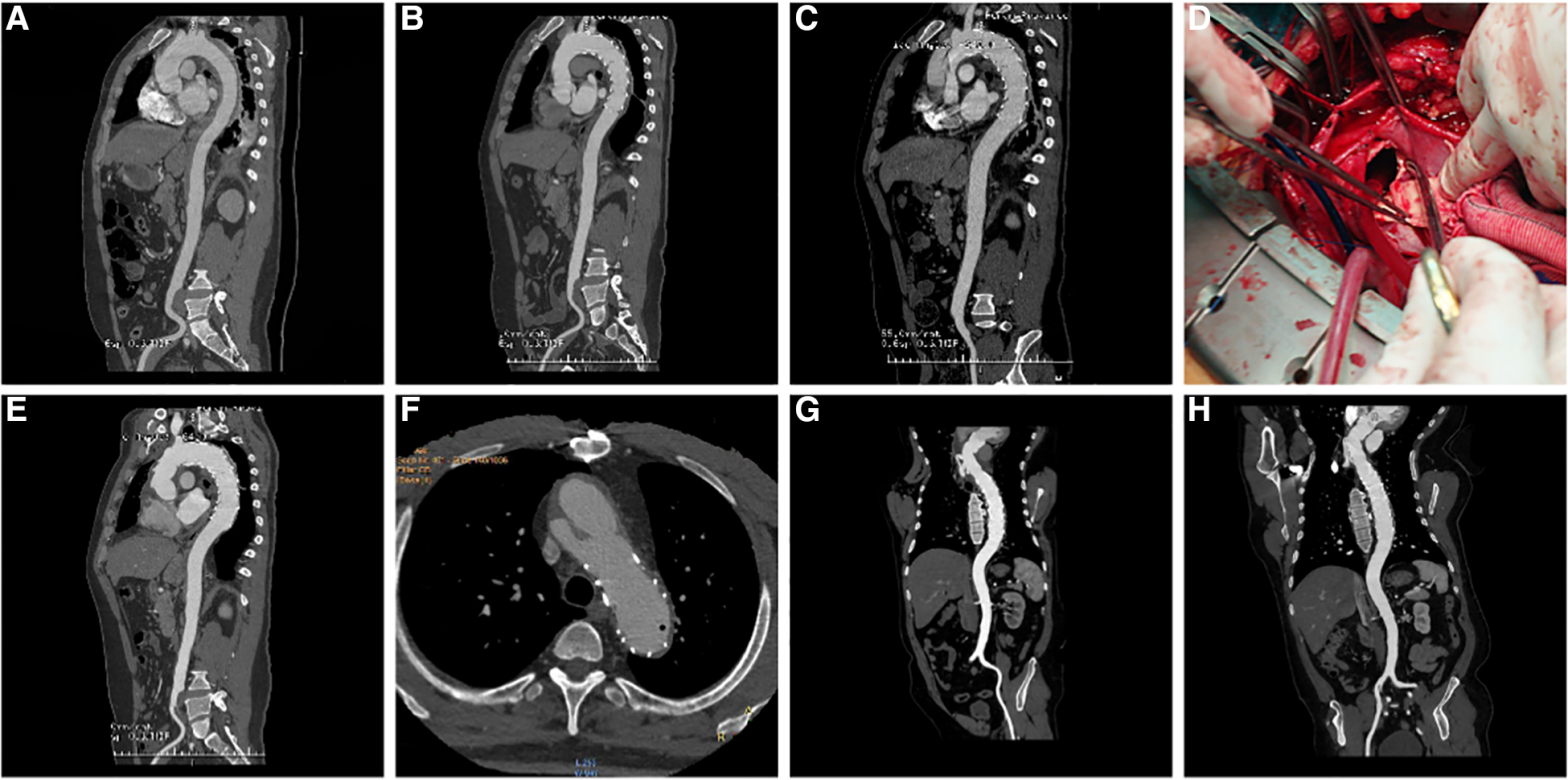
(**A**) three-dimensional (3D) reconstruction of preoperative surgery CTA showed intramural hematoma of descending aorta; (**B**) CTA demonstrated that the intramural hematoma was thinner than that before ten days following TEVAR; (**C**) five weeks following TEVAR, 3D reconstruction of CTA showed RTAD; (**D**) “ascending aortic replacement, hemi-arch replacement, and stented elephant trunk” was implemented in the emergency, and the entry tear was at the proximal stent; (**E**) CTA two weeks following the surgery showed changes in the ascending aorta and the arch after replacement; (**F**) At 90 months after the first surgery, local dissecting aneurysms at the arch were observed; (**G**) “ascending aortic replacement, total arch replacement, and stented elephant trunk” were performed during the second surgery; (**H**) CT re-examination on the nine months after second surgery. CTA, CT angiography; TEVAR, Thoracic Endovascular Aortic Repair; RTAD, Retrograde Type A Aortic Dissection; 3D, three dimension.

**Figure 3:**
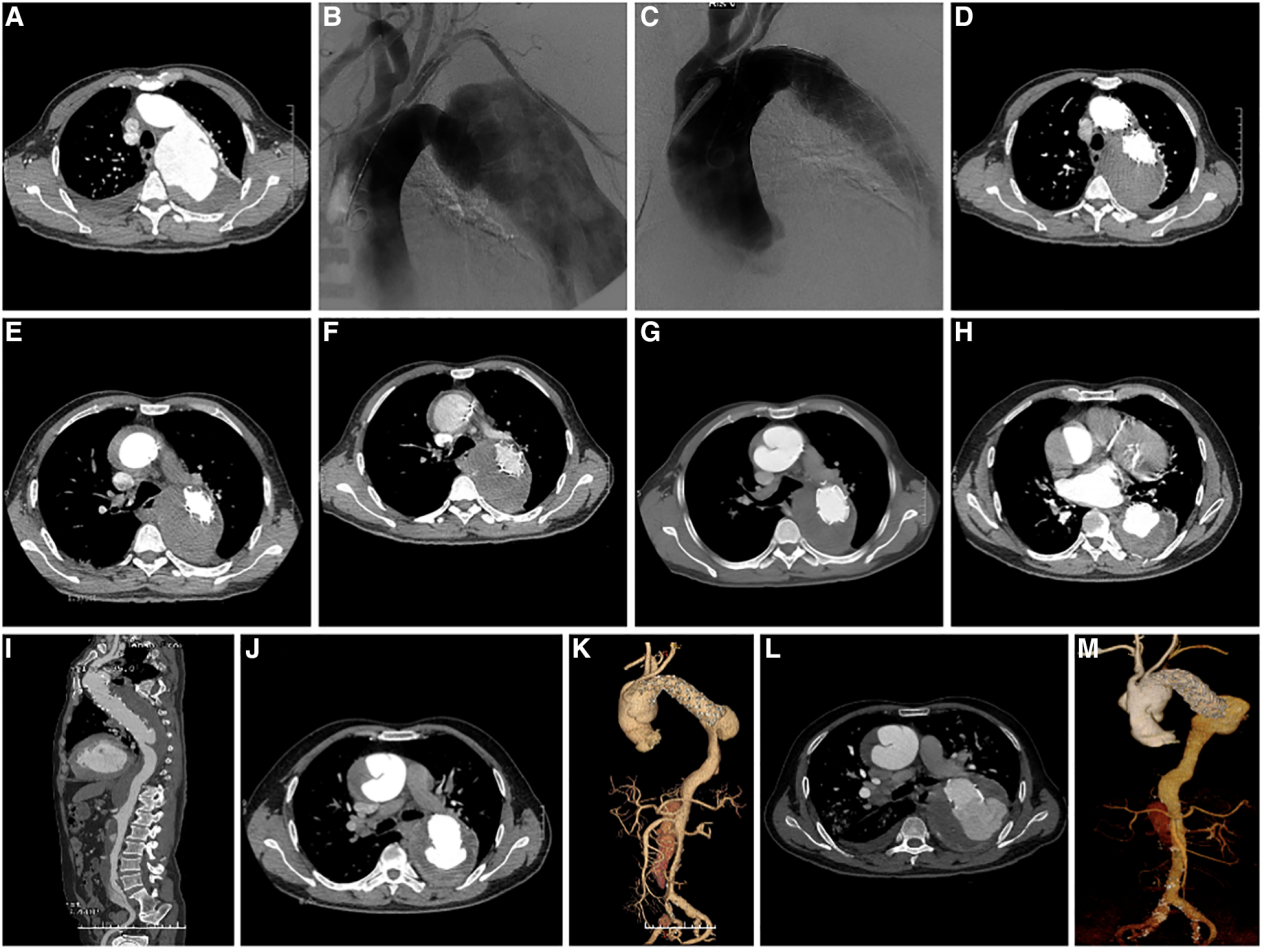
(**A**) preoperative CTA axial image of TEVAR showed TBAD; (**B**) Pre-stenting DSA shows a large false lumen with significant compression of the true cavity, and the entry located distal to the LSA; (**C**) DSA after stenting showed complete occlusion of the entry, widening of the true lumen, and improved blood flow; (**D**) CTA axial image ten days following TEVAR showed complete occlusion of the entry, thrombosis of the false lumen, and good visualization of the true lumen; (**E**) CTA at 13 days after TEVAR showed intermural hematoma formation in the ascending aorta; (**F**) CTA at three weeks after TEVAR showed intermural hematoma formation in the ascending aorta, with no significant change compared to the previous CTA; (**G**) CTA at 20 weeks after TEVAR showed progression of ascending aortic coarctation; (**H**) axial image of CTA at 32 months after TEVAR showed proximal RTAD and distal SINE; (**I**) 3D reconstruction of CTA at 32 months after TEVAR showed proximal RTAD and distal SINE; (**J**) axial image of CTA at 46 months after TEVAR showed progression of proximal RTAD and distal SINE. (**K**) 3D reconstruction of CTA at 46 months after TEVAR showed progression of both proximal RTAD and distal SINE; (**L**) Axial images of CTA at 56 months after TEVAR showed progression of both proximal RTAD and distal SINE; (**M**) 3D reconstruction of CTA at 56 months after TEVAR showed progression of both proximal RTAD and distal SINE. CTA, CT angiography; TEVAR, Thoracic Endovascular Aortic Repair; RTAD, Retrograde Type A Aortic Dissection; 3D, three dimension; SINE¸Stent Induced New Entry.

Among the 18 patients (13 males, five females; mean age, 56.78 years [range, 38–79 years]; Table 2). RTAD occurrences were observed at different time points. Specifically, ten patients with RTAD happened within the TEVAR perioperative period, with two cases during the surgery, six cases occurred within three months, two cases occurred after one year, and the longest interval being 72 months following TEVAR. The longest follow-up period was 130 months, and the shortest was only one day in RTAD group. Four patients were lost to the follow-up. Seven patients died during the follow-up period. TEVAR was initially implemented in 17 cases, while the operation procedure was temporarily altered in one case. The SG utilized comprised ten cases of Medtronic Vialiant, three cases of Cook Zenith, three cases of Gore TAG, and one case of Shanghai MicroPort Castor integrated branching stent. The position of the new entry in 13 RTAD patients was at the proximal region of the SG, and five instances were more than 2 cm distant from the proximal region of the SG. Besides, 17 cases were at the greater curvature of the aorta, and 1 case was at the lesser curvature. It is noteworthy that two cases developed RTAD during the operation. Although the initial surgical plan for case six was to perform a thoracotomy with ascending aorta IA/LCCA bypass and TEVAR, an interim and urgent change was made to perform ascending aortic replacement, total arch replacement with frozen elephant trunk. This decision was prompted by the presence of a dissection observed during the clamping of the ascending aortic wall while reconstructing the branches of the arch. This patient was discharged from the hospital following a satisfactory recovery and was lost to follow-up. In another case with intramural hematoma of TBAD, dilation of a narrow TAG stent with a GORE trilobate balloon resulted in a new entry at the greater curvature of the proximal region of the SG. As a remedial measure, the patient underwent rescue implantation of the second TAG stent after an emergency LCCA-LSA bypass. The patient showed good recovery and remained in a stable condition during the last follow-up. Six of the remaining 16 RTAD patients underwent successful surgical repairs. Case 2 with a favorable outcome and a follow-up of 130 months, was the only one that respectively had ascending aortic replacement, hemi-arch replacement with frozen elephant trunk and ascending aortic replacement, total arch replacement with frozen elephant trunk due to the pain in the chest and back at postoperative five weeks and 90 months (Figure 2). In 10 RTAD patients treated conservatively, seven deaths occurred, and three patients were lost to follow-up. Case one with ascending aorta hematoma formation but no clear entry tear developed RTAD in the perioperative period. At six months of follow-up, the ascending aortic entry tear was visible and located at the proximal portion of the SG. At 33 months of follow-up, stent induced new entry (SINE) occurred at the distal part of SG. At 46 months of follow-up, both proximal RTAD and distal SINE advanced. He died at 56 months due to acute left heart failure combined with mitral valve prolapse (Figure 3). The characteristics of 18 patients complicated with RTAD during or after TEVAR were presented in Table 2.

**Table 2:** Characteristics of 18 patients complicated with RTAD during or after TEVAR.

Cases	Age	Gender	Coexisting conditions	Stent graft	Oversizing ratio	Onset time	Location of new tear	Cause of RTAD	Treatment	Follow-up and outcome
1	58Y	M	Hypertension	MEDTRONIC VALIANT	10	12D	TSG	SG	Medical	56M(died)
2	38Y	M	Hypertension	MEDTRONIC VALIANT	10	5W	TSG	SG	Surgery	130M
3	48Y	F	Marfan	COOK Zenith	13	3M	TSG	SG	Medical	3M(lost)
4	43Y	M	–	MEDTRONIC VALIANT	6	72M	≥2 cm (TSG)	Progress	Surgery	111M
5	72Y	M	Hypertension	MEDTRONIC VALIANT	5	16D	≥2 cm (TSG)	Clamp	Medical	97M(died)
6	44Y	M	Hypertension	–	–	Intraoperative	Ascending Aorta	Clamp	Surgery	1M(lost)
7	66Y	M	Hypertension	COOK Zenith	10	3M	TSG	SG	Medical	3M(lost)
8	43Y	M	Hypertension	COOK Zenith	9	3M	≥2 cm (TSG)	Progress	Surgery	96M
9	79Y	F	Hypertension	GORE TAG	6	9D	TSG	SG	Medical	24M(died)
10	78Y	M	–	GORE TAG	11	1W	TSG	SG	Medical	6M(died)
11	43Y	M	–	GORE TAG	11	Intraoperative	TSG	Dilation	Surgery	46M
12	60Y	M	Hypertension	MEDTRONIC VALIANT	9	1W	TSG	SG	Medical	2D(died)
13	55Y	F	Hypertension	MEDTRONIC VALIANT	8	3M	TSG	SG	Surgery	37M
14	43Y	M	Hypertension	MEDTRONIC VALIANT	0	4W	TSG	SG	Surgery	36M
15	59Y	M	–	MEDTRONIC VALIANT	6	4D	TSG	SG	Medical	6D(lost)
16	62Y	F	Hypertension	MEDTRONIC VALIANT	2	9D	TSG	SG	Medical	2D(died)
17	55Y	M	Hypertension	MEDTRONIC VALIANT	3	11D	TSG	Dilation	Surgery	26M
18	76Y	F	Hypertension	Microport Castor	9	6W	≥2 cm (TSG)	Progress	Medical	1D(died)

M, Male; F, Female; Y, Year; M, Month; W, Week; D, Day; TSG, tip of stent graft; SG, Stent graft; RTAD, Retrograde type A aortic dissection; TEVAR, thoracic endovascular aortic repair.

### Univariate logistic regression analysis affecting the incidence of RTAD

There was a statistically significant association between the SG oversizing ratio (OR = 0.82, 95%CI = 0.73–0.93, *P* = 0.0011) and diameters of ascending aorta (OR = 1.09, 95%CI = 1.01–1.18, *P* = 0.0316) to the occurrence rate of RTAD. There was no statistically significant between the operation timing, the type of SG, medical history data, and operation mode were to the incidence of RTAD (*P* > 0.05). Full details are shown in Table 3.

**Table 3:** Univariate analysis of the variables for RTAD occurrence.

Variables	Value	RTAD occurrence	
		OR (95%CI)	*P*-value
Age	52.52 ± 12.71	1.03 (0.99, 1.06)	0.1553
Gender			
Female	260 (15.40%)	1.0	
Male	1,428 (84.60%)	0.47 (0.17, 1.33)	0.1531
Trauma			
No	1,631 (96.62%)	1.0	
Yes	57 (3.38%)	0.00 (0.00, Inf)	0.9870
Connective tissue disease			
No	1,649 (97.69%)	1.0	
Yes	39 (2.31%)	2.53 (0.33, 19.47)	0.3738
Hypertension			
No	422 (25.00%)	1.0	
Yes	1,266 (75.00%)	0.87 (0.31, 2.44)	0.7845
Diabetes			
No	1,670 (98.93%)	1.0	
Yes	18 (1.07%)	0.00 (0.00, Inf)	0.9888
Cardiovascular and cerebrovascular diseases			
No	1,469 (87.03%)	1.0	
Yes	219 (12.97%)	2.62 (0.92, 7.41)	0.0702
Renal insufficiency			
No	1,638 (97.04%)	1.0	
Yes	50 (2.96%)	0.00 (0.00, Inf)	0.9878
Smoking			
No	1,093 (64.75%)	1.0	
Yes	595 (35.25%)	1.85 (0.73, 4.69)	0.1946
Other complications			
No	1,396 (82.70%)	1.0	
Yes	292 (17.30%)	2.42 (0.90, 6.50)	0.0797
Pathological type			
TBAD	1,592 (94.31%)	1.0	
Non-TBAD	96 (5.69%)	0.00 (0.00, Inf)	0.9890
Pathological stage			
Acute	1,433 (84.89%)	1.0	
Chronic	255 (15.11%)	0.70 (0.16, 3.06)	0.6359
Surgical producers			
TEVAR	1,312 (77.73%)	1.0	
(Non-thoracotomy) Hybrid	149 (8.83%)	1.36 (0.30, 6.08)	0.6879
(Thoracotomy) Hybrid	73 (4.32%)	2.81 (0.62, 12.71)	0.1,785
TEVAR (Fenestration Technique)	52 (3.08%)	1.96 (0.25, 15.27)	0.5208
TEVAR (Branch Stent Repair Techniques)	102 (6.04%)	0.00 (0.00, Inf)	0.9888
Timing of surgical intervention			
Chronic phase	137 (8.12%)	1.0	
Subacute phase	156 (9.24%)	0.00 (0.00, Inf)	0.9858
Acute phase	1,395 (82.64%)	0.78 (0.18, 3.44)	0.7463
Mean time from disease onset to surgery	89.14 ± 470.25	1.00 (1.00, 1.00)	0.6847
Different stent design			
Poximal barbs		Ref	
Fully covered SG		2.2 (0.2, 21.9)	0.505
Proximal flared scallops or partially uncovered stents		0.4 (0.1, 1.8)	0.205
Proximal bare stent		0.5 (0.1, 2.0)	0.368
Oversizing ratio (%)	11.15 ± 4.77	0.82 (0.73, 0.93)	0.0011
Proximal landing zone			
Z0	203 (12.03%)	1.0	
Z1	436 (25.84%)	0.58 (0.15, 2.17)	0.4163
Z2	952 (56.43%)	0.42 (0.13, 1.41)	0.1618
Z3	96 (5.69%)	0.00 (0.00, Inf)	0.9886
Arch type**			
Type Ⅰ	639 (37.86%)	1	
Type Ⅱ	852 (50.47%)	0.86 (0.31, 2.37)	0.7647
Type Ⅲ	197 (11.67%)	1.40 (0.36, 5.45)	0.6311
Retrograde tear conditions			
No obvious retrograde tear	956 (56.64%)	1	
Retrograde tear to aortic arch	538 (31.87%)	1.79 (0.71, 4.54)	0.2198
Retrograde tear to ascending aorta	194 (11.49%)	0.00 (0.00, Inf)	0.9900
Diameter of ascending aorta (mm)	38.08 ± 4.96	1.09 (1.01, 1.18)	0.0316

SG, Stent graft; RTAD, retrograde type A aortic dissection; OR, odds ratio; CI, confidence interval.

** The classification of the aortic arch follows the methodology proposed by Myla.

### Multivariate logistic regression analysis affecting the incidence of RTAD

The results of multivariate logistic regression analysis following the adjustment for confounding factors showed that the oversizing ratio influenced the incidence of RTAD (*P* < 0.05). The diameter of the ascending aorta, on the other hand was not associated with RTAD (*P* > 0.05). Details are supplied in Table 4.

**Table 4:** Multivariate logistic regression analysis of stent graft oversizing ratio, ascending aortic diameter and incidence of RTAD.

Variables	RTAD			
	Non-adjusted OR (95%CI)	*P*-value	adjusted OR (95%CI)	*P*-value
SG oversizing ratio	0.83 (0.74, 0.94)	0.0026	0.83 (0.73, 0.94)	0.0028
Ascending aorta diameter	1.06 (0.97, 1.15)	0.1818	1.06 (0.96, 1.17)	0.2373

SG, Stent graft; RTAD, retrograde type A aortic dissection; OR, odds ratio; CI, confidence interval.

## Discussion

The occurrence of RTAD during or following TEVAR is rare but carries severe consequences (16–18). Wang et al. (8) noted in a meta-analysis that the overall incidence of RTAD was 2.2%. Eggebrecht et al. (2) reported an overall incidence of RTAD of 1.3% and a mortality rate of 42% in a multicenter retrospective study. Analyzing the statistical data of 1,688 TEVAR patients at our facility over the past 16 years, we found an overall incidence of RTAD at 1.1%, accompanied by a 39% all-cause mortality rate.

Is the design of the SG connected to the occurrence of RTAD? Dong et al. (7) reported 11 cases of proximal bare SG, nine cases of a new entry at the proximal region of the bare stent, and one case inside the anchoring area of the bare stent. Therefore, the authors concluded that the proximal bare stents were closely associated with the occurrence of RTAD. However, there is no consensus on this point of view. Ma et al. (13) hold the point that the radial force strength and the leverage effect of the SG rather than proximal bare SG were associated with RTAD. Ten patients in the current series had SG incorporated proximal bare metal stent, while the remaining seven had no proximal bare SG implanted, including three proximal barbs devices, three proximal flared scallop devices, and a covered debranching stent. The RTAD group consisted of patients who had a wide range of SG implanted and our statistical analysis indicated that the occurrence of RTAD was not directly linked to the stent design. It is worth noting that among the RTAD patients, three had Gore TAG stents with flared scallops, which were observed to have a significant abduction force when examined *in vitro*. To address this issue, a second-generation device called the Gore C-TAG (Comfortable TAG) was developed, where the proximal flared scallops of the SG were replaced with partially uncovered stents measuring 4–5 mm in length. This modification effectively reduced the abduction force of the proximal stent. Furthermore, the utilization of the Gore C-TAG in our department has significantly surpassed the usage of its previous generation counterparts. Notably, no cases of RTAD have been observed in patients treated with the C-TAG stent, which may be attributed to its improved compliance and reduced radial force. The compliance of a stent plays a crucial role in determining the risk of RTAD, as supported by several literatures (19–22).

Is there a link between the pathological nature of the disease and the development of RTAD? Dong et al. (7) have highlighted that Marfan syndrome is an important risk factor for the occurrence of RTAD. The pulsatile movement of the stent against the aortic wall during the cardiac cycle could cause damage to the aortic wall, leading to RTAD, particularly in patients with aortic dissection and connective tissue disorders such as Marfan syndrome. In our cohort, all 18 RTAD patients had aortic dissection, and one patient had Marfan syndrome. However, further evidence was needed to support the notion that aortic dissection was associated with RTAD than other thoracic aortic conditions.

Previous literature believed that a greater oversizing ratio was related to a higher RTAD. Kpodonu et al. (23) conducted a series of investigations involving seven cases with RTAD. Among these cases, two had a SG oversizing ratio close to 20%, and three had an oversizing ratio exceeding 20%. The study concluded that when the SG oversizing ratio surpasses 20%, the excessive radial force exerted on the intima may lead to intimal damage, potentially causing RTAD. Similarly, academics considered that 10%–15% of the SG oversizing ratio is sufficient and that excessive SG oversizing ratio should be avoided to prevent RTAD (7). However, Holger et al. (2) put forward different viewpoints and reported a multicenter study of 48 cases of RTAD with an average SG oversizing ratio of 6%. Among the 18 RTAD patients, the average oversizing rate was 7.5%. Notably, 82% of these cases fell within the range of 10% oversizing, with only three cases (18%) exceeding this threshold. The statistical analysis demonstrated a significantly higher incidence of RTAD in TEVAR patients with stent oversizing less than 10% compared to those with stent oversizing greater than 10%. This finding suggests that the presence of a certain gap, commonly referred to as a “bird beak” between the stent and the vessel wall may contribute to the up and down movement of the stent with each cardiac cycle, directly leading to RTAD. In our experience, appropriately increasing the SG oversizing ratio, especially for the Gore stent, could indeed reduce the “bird beak” phenomenon. It is worth drawing attention to that RTAD was not detected in 60 patients with SG oversizing greater than 20%. However, the sample size might have been too small to depict reality.

According to Canaud et al. (24), the proximal sealing zone in the aortic arch is one of the risk factors for the occurrence of RTAD. In our study, 96.8% RTAD patients had involvement in the Z0–2 region, but there was no significant difference in the incidence of RTAD between the Z0–2 and Z3–4 regions. With the extensive application of fenestration and debranching techniques in clinical practice, manipulation of the arch undoubtedly raises the frequency of RTAD (22, 25–27).

The diameter of the ascending aorta is also linked to the presence of RTAD. Williams et al. (10) proposed that the ascending aorta diameter exceeding 40 mm is a risk factor for the occurrence of RTAD, which contradicts our findings. Notably, two patients with ascending aorta diameters greater than 40 mm in the RTAD group were caused by iatrogenic factors. Therefore, caution should be taken when undertaking hybridization to avoid RTAD (6, 28, 29).

It is reported that surgical procedures were accountable for approximately 5% of aortic dissections (30, 31). Although the results of the present study showed that different surgical timing and methods did not directly affect the occurrence of RTAD, RTAD may be induced in certain specific surgical procedures such as balloon dilation. Two RTAD patients with aortic dissection in this research were associated with balloon dilation, and one of them with new dissection formation when an inadequately deployed stent in descending aortic arch was dilated by balloon. In another patients, chimney stent implantation of the LCCA and balloon dilation were performed urgently due to accidental stent displacement that covered the LCCA. Although no abnormalities were detected during the TEVAR procedure, a significant bouncing movement of the bare stent was observed during balloon expansion of the chimney stent when reviewing intraoperative angiography. This vigorous movement of the stent has the potential to damage the vascular wall and contribute to the occurrence of RTAD. Impressively, individuals with thoracic aortic aneurysm and aortic coarctation did not experience RTAD after balloon dilation in the current study. Therefore, balloon dilation should be avoided in patients with aortic dissection.

Gender, age, comorbidities, and arch type were not shown to be directly connected to the occurrence of RTAD in this research. It is worth noting that 94% of patients in this research have an aortic dissection, and the average age is 52 years old, which is 10–15 years younger than the average age reported in Europe and the United States. Additionally, these patients had fewer underlying diseases, which could be one of the reasons for the relatively low overall incidence of RTAD (32, 33).

So, how could RTAD be effectively avoided? (I) If TEVAR is selected for aortic dissection patients with connective tissue disease, emphasis should be made on operational issues such as avoiding unnecessary operations in the arch and selecting stent grafts with higher flexibility; (II) The right SG oversizing might assist the stent in conforming better with the aortic wall; (III) When the diameter of the ascending aorta exceeds 40 mm, especially in the presence of calcification and other abnormalities, it is advisable to avoid surgical intervention in the ascending aorta. If circumstances permit, simultaneous replacement of the ascending aorta is a preferred alternative; (IV) Balloon dilation was not recommended for patients with aortic dissection during TEVAR. This study offered objective data on the rate of RTAD utilizing a large sample size from a single center. However, it has to be mentioned that the primary limitation of this study is its retrospective characteristic.

## Conclusion

RTAD is a rare yet catastrophic complication. It could occur both during the procedure, early and late postoperative periods. Maintaining an appropriate SG oversizing ratio is crucial to minimize the risk of RTAD.

## Data Availability

The raw data supporting the conclusions of this article will be made available by the authors, without undue reservation.

## References

[1] Bicknell C Powell JT. Aortic disease: thoracic endovascular aortic repair. Heart. (2015) 101(8):586–91. 10.1136/heartjnl-2014-306690 25678497

[2] Eggebrecht H Thompson M Rousseau H Czerny M Lönn L Mehta RH Retrograde ascending aortic dissection during or after thoracic aortic stent graft placement: insight from the European registry on endovascular aortic repair complications. Circulation. (2009) 120(11 Suppl):S276–81. 10.1161/CIRCULATIONAHA.108.835926 19752379

[3] Erbel R Aboyans V Boileau C Bossone E Bartolomeo RD Eggebrecht H 2014 ESC guidelines on the diagnosis and treatment of aortic diseases: document covering acute and chronic aortic diseases of the thoracic and abdominal aorta of the adult. The task force for the diagnosis and treatment of aortic diseases of the European society of cardiology (ESC). Eur Heart J. (2014) 35(41):2873–926. 10.1093/eurheartj/ehu281 25173340

[4] İslim F Erbahçeci Salık A Güven K Bakuy V Çukurova Z. Endovascular repair of thoracic and abdominal aortic ruptures: a single-center experience. Diagn Interv Radiol. (2014) 20(3):259–66. 10.5152/dir.2013.13165 24412816 PMC4463350

[5] Fossaceca R Guzzardi G Cerini P Parziale G Stanca C Micalizzi E Endovascular treatment of thoracic aortic aneurysm: a single-center experience. Ann Vasc Surg. (2013) 27(8):1020–8. 10.1016/j.avsg.2012.07.032 23790762

[6] Higashigawa T Kato N Chino S Hashimoto T Shimpo H Tokui T Type A aortic dissection after thoracic endovascular aortic repair. Ann Thorac Surg. (2016) 102(5):1536–42. 10.1016/j.athoracsur.2016.04.024 27316317

[7] Dong ZH Fu WG Wang YQ Guo DQ Xu X Ji Y Retrograde type A aortic dissection after endovascular stent graft placement for treatment of type B dissection. Circulation. (2009) 119(5):735–41. 10.1161/CIRCULATIONAHA.107.759076 19171859

[8] Wang L Zhao Y Zhang W Shu X Wang E Guo D Retrograde type A aortic dissection after thoracic endovascular aortic repair: incidence, time trends and risk factors. Semin Thorac Cardiovasc Surg. (2021) 33(3):639–53. 10.1053/j.semtcvs.2020.11.010 33181306

[9] Yammine H Briggs CS Stanley GA Ballast JK Anderson WE Nussbaum T Retrograde type A dissection after thoracic endovascular aortic repair for type B aortic dissection. J Vasc Surg. (2019) 69(1):24–33. 10.1016/j.jvs.2018.04.047 30580780

[10] Williams JB Andersen ND Bhattacharya SD Scheer E Piccini JP McCann RL Retrograde ascending aortic dissection as an early complication of thoracic endovascular aortic repair. J Vasc Surg. (2012) 55(5):1255–62. 10.1016/j.jvs.2011.11.063 22265798 PMC3699184

[11] Dong Z Fu W Wang Y Wang C Yan Z Guo D Stent graft-induced new entry after endovascular repair for Stanford type B aortic dissection. J Vasc Surg. (2010) 52(6):1450–7. 10.1016/j.jvs.2010.05.121 20800417

[12] Gorlitzer M Weiss G Moidl R Folkmann S Waldenberger F Czerny M Repair of stent graft-induced retrograde type A aortic dissection using the E-vita open prosthesis. Eur J Cardiothorac Surg. (2012) 42(3):566–70. 10.1093/ejcts/ezs041 22371519

[13] Ma T Dong ZH Fu WG Guo DQ Xu X Chen B Incidence and risk factors for retrograde type A dissection and stent graft-induced new entry after thoracic endovascular aortic repair. J Vasc Surg. (2018) 67(4):1026–33.e2. 10.1016/j.jvs.2017.08.070 29097043

[14] Xu SD Huang FJ Yang JF Li ZZ Yang S Du JH Early and midterm results of thoracic endovascular aortic repair of chronic type B aortic dissection. J Thorac Cardiovasc Surg. (2010) 139(6):1548–53. 10.1016/j.jtcvs.2009.08.051 19910003

[15] Xu SD Huang FJ Yang JF Li ZZ Wang XY Zhang ZG Endovascular repair of acute type B aortic dissection: early and mid-term results. J Vasc Surg. (2006) 43(6):1090–5. 10.1016/j.jvs.2005.12.070 16765220

[16] Li YL Ye JC Yancu H Liu B Wang YZ Wang WJ Thoracic endovascular aortic repair for type B aortic dissection associated with retrograde type A intramural hematoma. J Vasc Interv Radiol. (2020) 31(8):1334–41. 10.1016/j.jvir.2020.01.017 32127315

[17] Huang CY Weng SH Weng CF Chen WY Chen IM Hsu CP Factors predictive of distal stent graft-induced new entry after hybrid arch elephant trunk repair with stainless steel-based device in aortic dissection. J Thorac Cardiovasc Surg. (2013) 146(3):623–30. 10.1016/j.jtcvs.2012.07.052 23040193

[18] Wang G Zhai S Li T Shi S Zhang Z Liang K Mechanism and management of retrograde type A aortic dissection complicating TEVAR for type B aortic dissection. Ann Vasc Surg. (2016) 32:111–8. 10.1016/j.avsg.2015.09.028 26806250

[19] Lu S Lai H Wang C Sun X Hong T Song K Surgical treatment for retrograde type A aortic dissection after endovascular stent graft placement for type B dissection. Interact Cardiovasc Thorac Surg. (2012) 14(5):538–42. 10.1093/icvts/ivs043 22361126 PMC3329296

[20] Dun Y Shi Y Guo H Liu Y Zhang B Sun X The surgical management of retrograde type A aortic dissection after thoracic endovascular aortic repair. Interact Cardiovasc Thorac Surg. (2020) 30(5):732–8. 10.1093/icvts/ivz326 32016403

[21] Chen Y Zhang S Liu L Lu Q Zhang T Jing Z. Retrograde type A aortic dissection after thoracic endovascular aortic repair: a systematic review and meta-analysis. J Am Heart Assoc. (2017) 6(9):e004649. 10.1161/JAHA.116.004649 28939705 PMC5634245

[22] Idrees J Arafat A Johnston DR Svensson LG Roselli EE. Repair of retrograde ascending dissection after descending stent grafting. J Thorac Cardiovasc Surg. (2014) 147(1):151–4. 10.1016/j.jtcvs.2013.08.075 24139893

[23] Kpodonu J Preventza O Ramaiah VG Shennib H Wheatley GH 3rd Rodriquez-Lopez J Retrograde type A dissection after endovascular stenting of the descending thoracic aorta. Is the risk real? Eur J Cardiothorac Surg. (2008) 33(6):1014–8. 10.1016/j.ejcts.2008.03.024 18424065

[24] Canaud L Ozdemir BA Patterson BO Holt PJ Loftus IM Thompson MM. Retrograde aortic dissection after thoracic endovascular aortic repair. Ann Surg. (2014) 260(2):389–95. 10.1097/SLA.0000000000000585 24441822

[25] Masuda T Hata M Yamaya K Suzuki T Terao N. Two cases of endovascular repair with the stent graft for retrograde type A acute aortic dissection with complications. Ann Thorac Cardiovasc Surg. (2019) 25(5):278–82. 10.5761/atcs.cr.17-00200 29503377 PMC6823173

[26] Tsai CL. The “lantern” procedure to simplify treatment of retrograde type A dissection after thoracic endograft stenting. Ann Thorac Surg. (2016) 101(4):e129–31. 10.1016/j.athoracsur.2015.10.079 27000619

[27] Robertson LC Holdaway D Yap CH. Retrograde type A aortic dissection treated with continuous perfusion “branch-first” aortic arch replacement technique. Heart Lung Circ. (2015) 24(12):e206–9. 10.1016/j.hlc.2015.07.004 26422534

[28] Shetty V Vohra HA Viola N Brown I Langley SM. Surgical intervention for retrograde type A aortic dissection caused by endovascular stent insertion for type B aortic dissection. J Thorac Cardiovasc Surg. (2008) 135(6):1387–8. 10.1016/j.jtcvs.2007.11.047 18544393

[29] Li B Pan XD Ma WG Zheng J Liu YL Zhu JM Stented elephant trunk technique for retrograde type A aortic dissection after endovascular stent graft repair. Ann Thorac Surg. (2014) 97(2):596–602. 10.1016/j.athoracsur.2013.09.033 24210620

[30] Dobrilovic N Arslan B McCarthy WJ March RJ Turba UC Michalak L Delayed retrograde ascending aortic dissection after endovascular repair of descending dissection. Ann Thorac Surg. (2016) 101(6):2357–8. 10.1016/j.athoracsur.2015.06.109 27211942

[31] Gandet T Canaud L Ozdemir BA Ziza V Demaria R Albat B Factors favoring retrograde aortic dissection after endovascular aortic arch repair. J Thorac Cardiovasc Surg. (2015) 150(1):136–42. 10.1016/j.jtcvs.2015.03.042 25936469

[32] Mamopoulos AT Nowak T Luther B. Retrograde ascending Stanford B aortic dissection complicating a routine infrarenal endovascular aortic reconstruction. J Vasc Surg. (2013) 58(1):208–11. 10.1016/j.jvs.2012.10.111 23352359

[33] Liu Z Zhang Y Liu C Huang D Zhang M Ran F Treatment of serious complications following endovascular aortic repair for type B thoracic aortic dissection. J Int Med Res. (2017) 45(5):1574–84. 10.1177/0300060517708893 28701057 PMC5718725

